# A phase conversion method to anchor ZIF-8 onto a PAN nanofiber surface for CO_2_ capture

**DOI:** 10.1039/d1ra06480k

**Published:** 2021-12-23

**Authors:** Zeyu Li, Zhejian Cao, Carlos Grande, Wenjing Zhang, Yibo Dou, Xiaosen Li, Juan Fu, Nasir Shezad, Farid Akhtar, Andreas Kaiser

**Affiliations:** Department of Energy Conversion and Storage, Technical University of Denmark (DTU) Denmark akai@dtu.dk; Department of Engineering Sciences and Mathematics, Luleå University of Technology (LTU) Sweden; Process Technology, SINTEF Norway; Department of Environmental Engineering, Technical University of Denmark (DTU) Denmark; Guangzhou Center for Gas Hydrate Research, Chinese Academy of Sciences (CAS) China

## Abstract

Polyacrylonitrile (PAN) nanofibers were prepared by electrospinning and coated with zeolitic imidazolate framework-8 (ZIF-8) by a phase conversion growth method and investigated for CO_2_ capture. The PAN nanofibers were pre-treated with NaOH, and further coated with zinc hydroxide, which was subsequently converted into ZIF-8 by the addition of 2-methyl imidazolate. In the resulting flexible ZIF-8/PAN composite nanofibers, ZIF-8 loadings of up to 57 wt% were achieved. Scanning electron microscopy and energy-dispersive X-ray spectroscopy (EDS) showed the formation of evenly distributed submicron-sized ZIF-8 crystals on the surface of the PAN nanofibers with sizes between 20 and 75 nm. X-ray photoelectron spectroscopy (XPS) and carbon-13 nuclear magnetic resonance (^13^C NMR) investigations indicated electrostatic interactions and hydrogen bonds between the ZIF-8 structure and the PAN nanofiber. The ZIF-8/composite nanofibers showed a high BET surface area of 887 m^2^ g^−1^. CO_2_ adsorption isotherms of the ZIF-8/PAN composites revealed gravimetric CO_2_ uptake capacities of 130 mg g^−1^ (at 298 K and 40 bar) of the ZIF-8/PAN nanofiber and stable cyclic adsorption performance.

## Abstract 2

Poly acryl nitrile (PAN) nanofibers were prepared by electrospinning and coated with zeolitic imidazolate framework-8 (ZIF-8) by a phase conversion growth method and investigated for CO_2_ capture.

## Graphical abstract

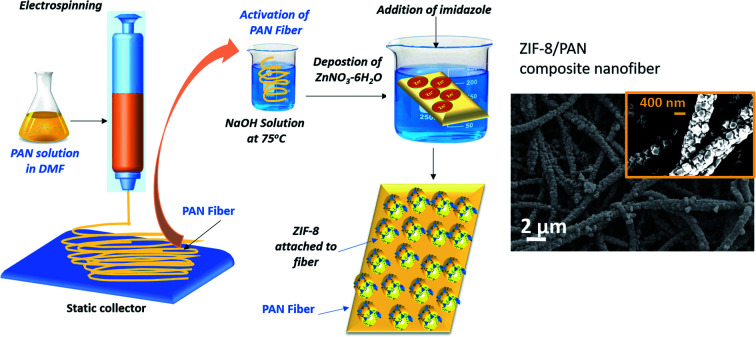

## 1. Introduction

Recently, the NET-ZERO 2050 report was published by the European Climate Foundation,^1^ which pointed out the urgency to achieve zero carbon emission by 2050 by making the energy system and transportation more sustainable and energy-efficient with the goal to eliminate carbon emissions. A part of the activities to achieve these goals is more efficient gas separation, capture and storage technologies for carbon capture and storage (CCS). Adsorption-based methods, such as pressure swing adsorption (PSA)^2^ or temperature swing adsorption (TSA)^3^ technologies offer opportunities for tailor-made gas separation of CO_2_ from gas mixtures^4^ and subsequent storage, for example in biogas upgrading^5^ and storage as biomethane (CH_4_).^6^ CH_4_ produced from sustainable sources such as biogas, can help to reduce anthropogenic CO_2_ emissions. One key point to improve adsorption-based technologies is exploring and improving advanced microporous materials (sorbents),^7^ such as zeolites,^8^ carbon molecular sieves^9^ and active carbon.^10^ It has been shown that by heteroatom-doping the CO_2_ adsorption can be significantly affected (*e.g.* in activated carbons) by creating highly polar adsorption sites for the CO_2_ molecule and Lewis acid–base interactions. This usually results in improved selectivities in CO_2_ separation.^11–13^ Another promising new class of materials are metal–organic frameworks (MOFs), which are assembled from metal ions/clusters and organic linkers.^14^ These materials can be prepared from a variety of chemical compositions, resulting in structures with ultrahigh surface area of several thousand m^2^ g^−1^, large pore volumes with tailorable pore size and specific chemical sites for capturing guest molecules by adsorption. In these materials, the extremely large internal surface area and pore channels can be modified to introduce specific surface charges, hydrophilicity/hydrophobicity or uniformity of pore channels. Zeolitic Imidazolate Frameworks (ZIFs), a sub-class of MOFs, have been proposed for gas separation and storage applications.^15^ These materials have first been synthesized by Yaghi's group.^16^ The structure consists of zinc(ii) cations and 2-methylimidazole anions (2-mim). Within the class of MOF materials, ZIFs have an interesting framework structure with high thermal and chemical stability. According to a report from Park *et al.*,^15^ ZIF-8 has a large sodalite (SOD) zeolite-like cavity (11.6 Å) and smaller apertures (3.4 Å). The size of these pores, the high achievable specific surface area of more than 1000 m^2^ g^−1^, and large micropore volumes indicate that this material has promising potential for CO_2_ capture and separation.

Beneath shaping of microporous materials into useful structures, such as pellets, granules or extrudates,^30^ recently, new approaches have been proposed to structure adsorbents (carbons, zeolites, MOFs *etc.*) into nanofibers to enhance gas transport,^17^ adsorption capacity^18^ and adsorption kinetics.^19^ For example, zeolite nanofibers of ZSM-5 have been fabricated by electrospinning, post shaping and carbonization of polyvinylpyrrolidone (PVP)^20^ the performance has been evaluated for CO_2_ separation from CO_2_/CH_4_ mixtures in synthetic biogas mixtures.

Due to the temperature instability of MOF materials, this approach to fabricate MOF-nanofiber composite materials is not feasible for the majority of MOF-based nanofibers. However, different approaches for the structuring of MOF-nanofiber materials for energy and environmental applications have been recently reviewed by Dou *et al.*,^21^ including *in situ* growth of MOF nanocrystals on the surface of polymer nanofibers. The preparation of MOF-polymer nanofibers through electrospinning is an elegant way to shape MOF materials into hierarchical porous nanofibrous sorbent materials at mild conditions with large surface-to-volume ratio, tailored pore size, and high permeability for gas separation processes. In this study we propose a phase inversion method to grow ZIF-8 on the surface of electrospun polyacrylonitrile (PAN) nanofibers for CO_2_ capture. We investigated the influence of the growth of the ZIF-8 nanoparticles on PAN nanofibers as a function of reaction time to maximize the CO_2_ gas uptake, which was expected to increase with the amount of the microporous ZIF-8 sorbent loaded on the inactive PAN nanofiber as backbone.

The resulting ZIF-8/PAN composite nanofibers have been characterized by scanning electron microscope-energy dispersive X-ray spectroscopy (SEM-EDS), X-ray diffraction pattern (XRD), surface area and pore size distribution analysis and by more advanced characterization techniques, such as X-ray photoelectron spectroscopy (XPS) and nuclear magnetic resonance spectroscopy (NMR).

Finally, the CO_2_ uptake of the ZIF-8/PAN composite nanofibers have been tested at pressures up to 40 bars and the cyclic adsorption–desorption properties of the composites have been investigated.

## 2. Experimental

### 2.1 Materials

For the *in situ* growth of ZIF-8, the raw chemicals zinc nitrate hexahydrate (Zn(NO_3_)_2_·6H_2_O), sodium hydroxide (NaOH) and 2-methylimidazole (2-mim) with purity over 99.0% were purchased from Sigma-Aldrich. For the electrospinning (ES), polyacrylonitrile (PAN) with an average molecular weight of 150 000 was chosen as polymer with a purity of 99.0%. The PAN was purchased from Parchem fine & specialty Co., Ltd *N*,*N*-Dimethylformamide (DMF) with purity of 99.0% was purchased from Sigma-Aldrich. Besides, the solvent of methanol and ethanol with purity of 99.9% were purchased from Sigma-Aldrich. The deionized water (D.I.W) was produced in the laboratory by an ultrapure system with a resistivity of 18.0 mΩ cm^−1^. All chemicals used in this work have not been further treated or purified. High purity gases of CO_2_ (99.99 mol%), CH_4_ (99.99 mol%), He (99.99 mol%) and N_2_ (99.99 mol%) (Foshan Huate Gas Co., Ltd, China) were used for gas adsorption experiments.

### 2.2 Preparation of ZIF-8/PAN composite nanofibers

The preparation of the ZIF-8/PAN composite nanofibers can be divided into four steps: in the 1^st^ step, the PAN solution was electrospun into PAN nanofibers. In the 2^nd^ step, the PAN nanofibers were immerged into a heated sodium hydroxide solution (75 °C) to activate the surface. In the 3^rd^ step, the metal-contained (Zn^2+^) precursors were attached on the active PAN surface. In the last step, the *in situ* growth process of ZIF-8 was initiated by the addition of the complementary precursor (2-mim solution).

For the preparation of the PAN nanofibers, 1 g PAN and 10 g DMF were mixed and sealed into a 50 mL plastic bottle with cylindrical zirconia balls to ball-mill by regular ball milling at 50 rpm for 24 hours. Then the dispersion solution was electrospun by needle-based electrospinning equipment (Linari s.r.l) with the voltage of 30 kV and a distance of 140 mm between the electrospinning needle and receiving substrate to prepare PAN nanofibers.

For the activation of the PAN nanofiber surface with hydroxide groups, 0.5 g of PAN nanofibers were immerged into the 50 mL NaOH-D.I.W solution (60 g NaOH dissolved into 500 mL D.I.W) and heated at 75 °C for 20 min to activate the fiber surface. The treated PAN nanofibers were 3 times washed with ethanol and dried afterwards in air at room temperature.

For the application of the metal precursor, the surface-treated PAN nanofibers were immersed into 100 mL of a 120 millimole per liter (mM) Zn(NO_3_)_2_ solution for 20 min to form Zn(OH)_2_ on the surface of PAN nanofibers. The applied Zn(NO_3_)_2_ solution consisted of 6.29 g Zn(NO_3_)_2_·6H_2_O in 180 mL of a 1 : 1 volumetric solvent mixture of methanol and ethanol.

For the final *in situ* growth process, 100 mL 480 mM 2-mim solution was added to the Zn(NO_3_)_2_ solution directly, where the Zn(OH)_2_-coated PAN fiber had been immerged already to complete the *in situ* growth process. Every 30 minutes, the samples were taken and dried after ethanol washing to identify the optimum loading and structure of ZIF-8 in the composite. The applied 2-mim solution consisted of 7.49 g 2-methylimidazole in 190 mL of a 1 : 1 volumetric solvent mixture of methanol and ethanol. According to reaction time the samples were labelled ZIF-8/PAN-*X* (with *X* resembling 30, 60 and 90 minutes of reaction time, see Table 1 further below).

**Table 1:** Summary of textural properties and crystallite size of ZIF-8/PAN composites

Sample ID	Average crystal size (nm)	Average pore size (Å)	BET surface area (m^2^ g^−1^)	Total pore volume (cm^3^ g^−1^)	Micro pore volume^a^ (cm^3^ g^−1^)
ZIF-8 powder	101	13.8	1015	0.70	0.51
ZIF-8/PAN-30	20	15.2	495	0.38	—
ZIF-8/PAN-60	36	13.0	862	0.56	—
ZIF-8/PAN-90	76	11.2	888	0.50	0.31

a The micro-pore volumes have been calculated from a T-plot, following a method reported by Galarneau *et al.*^22^

### 2.3 Preparation of ZIF-8 nanopowder

For the synthesis of ZIF-8 nanocrystals in powder form, 50 mL 120 mM Zn(NO_3_)_2_ solution and 50 mL 480 mL 2-mim solution were prepared (as described in Section 2.2) and mixed by vigorously magnetic stirring for 1 hour at room temperature, then the suspension solution was centrifuged to obtain the ZIF-8 colloids. Followed, the ZIF-8 colloids were washed with ethanol for 3 times. Finally, the ZIF-8 nanocrystals were dried at 100 °C to remove the residual ethanol, methanol and moisture.

### 2.4 Characterization of the materials

Scanning electron microscope-energy dispersive X-ray spectroscopy (SEM-EDS) were performed by a Zeisis Merlin FEGSEM, the samples were sputter-coated with the thin layer of the gold (around 12–16 nm).

X-ray diffraction (XRD) measurements were performed by a Rigaku Smartlab X-ray diffractometer, which was operated at 40 kV and 30 mA using Cu Kα radiation with step speed of 5° min^−1^. All of the samples were attached on a silicon-substrate sample holder. The miller indexes were identified by Match! 3 software.

X-ray photoelectron spectroscopy (XPS) was performed in the range of 200 to 1200 eV with ZIF-8 nanocrystals, pure PAN fiber and ZIF-8/PAN composite nanofibers in a angle resolved XPS chamber for depth profiling of surfaces (equipment from Thermo Scientific Nexsa).

^13^C nuclear magnetic resonance spectroscopy (NMR) was performed on a Bruker Avence III 300 Wide Bore solid state NMR spectrometer, using a 4 mm probe. The frequency of ^13^C was 75 MHz with the pulse program of CP-Toss, using 4 mm rotors at a MAS spinning frequency of 6500 Hz. The 90° ^13^C pulse-length was 4 μs, and pulse length in decoupling sequence is 7. Recycle delay is 5 s, while the sweep wide is 30 241.94 Hz. The carbon resonance line of adamantane was used as an external chemical shift standard and was assigned a value of 38.48 ppm. The scans for CP-Toss experiments was 1k. Besides, the samples were frozen and milled into powder with help of liquid nitrogen before it was filled into NMR tube.

The surface area as well as pore-related information (size distribution, total pore volume, micro-pore volume) determination of ZIF-8/PAN composite materials and ZIF-8 powders were measured by nitrogen adsorption isotherms at −196 °C (Autosorb-iQ-2, specific analysis for microstructure). The CO_2_ uptake measurements were also carried out in this equipment. All samples were degassed to below 10^−6^ bar and 383 K for 8 hours prior to the measurement.

The high-pressure CO_2_ adsorption performance was measured by an IsoSORP adsorption analyzer (TA instruments, New Castle, DE, USA), on samples with a weight of about 0.5 g. All samples were degassed at 110 °C under high vacuum for 4 h, following a buoyancy test with helium at 22 °C to determine the mass and volume of each sample. Then, the CO_2_ adsorption–desorption isotherms of the obtained ZIF-8/PAN composite were measured with CO_2_ dosing from high vacuum to 40 bar, then to high vacuum with an equilibrium point every 10 bar. The equilibrium for each step was set until the deviation of mass was less than 0.1 mg per 10 min.

## 3. Result and discussion

The *in situ* growth of the ZIF-8 on the surface of electrospun PAN nanofibers resulted in flexible, paper-like ZIF-8/PAN composite nanofiber mats with light yellow colour, whereas the solvothermally synthesized ZIF-8 powder showed white nanocrystals.

### 3.1 Microstructure of ZIF-8/PAN nanofiber structures

**Fig. 1:**
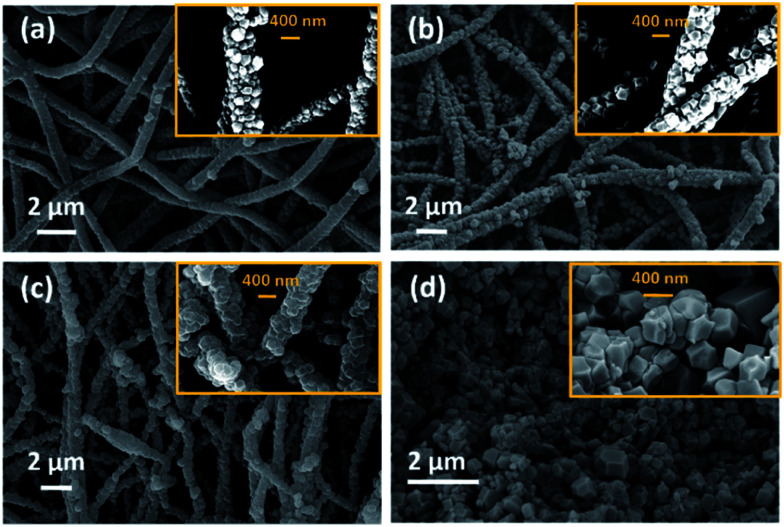
Scanning electron microscopy (SEM) images of ZIF-8/PAN composite nanofibers prepared by *in situ* growth and sampled after different reaction times of: 30 min (a), 60 min (b), 90 min (c) and a ZIF-8 nanopowder (d), synthesized under similar solvothermal conditions.

**Fig. 2:**
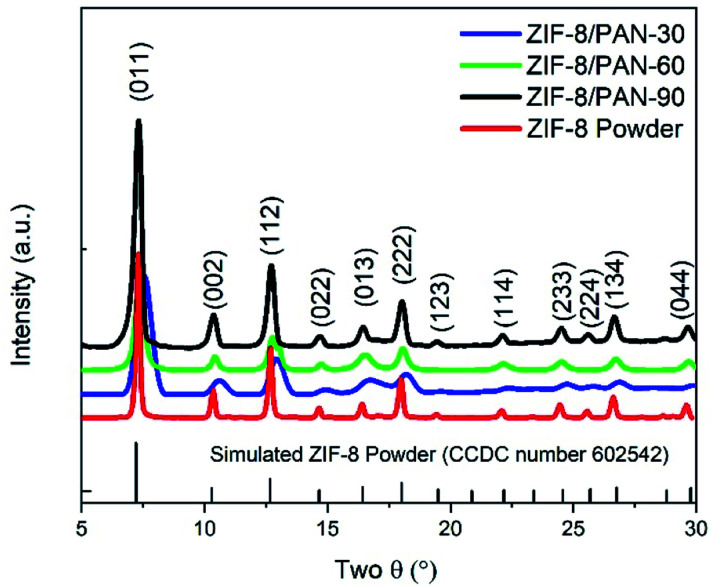
X-ray diffractograms of ZIF-8/PAN composites and a solvothermally synthesized ZIF-8 powder.

Fig. 1 shows scanning electron microscope (SEM) images of the different ZIF-8/PAN composite nanofibers after different *in situ* growth durations of 30 min (Fig. 1a), 60 min (Fig. 1b) and 90 min (Fig. 1c). Small ZIF-8 nanoparticles (with rhombic dodecahedral form) have evenly grown on the surface of the PAN nanofibers, forming a core–shell structure. Visual inspection indicate a good mechanical adhesion of the ZIF-8 crystal on the PAN polymer surface and this has also been observed in previous studies on ZIF-8/nanofiber composites and nanocrystals.^23–25^ X-ray diffractograms of the ZIF-8/PAN composites after 30, 60, 90 minutes reaction time are given in Fig. 2 together with the solvothermally synthesized ZIF-8 nanopowder.

The pattern-ray diffractogram of the synthesized ZIF-8/PAN composite nanofibers matched closely with the simulated X-ray diffractogram of ZIF-8 powder, which can be assigned to cubic *I*4̄3*m* space group,^15^ confirming the growth of ZIF-8 nanoparticles on the surface of PAN nanofibers. Lower relative diffraction intensity of X-rays and broadening of diffraction peaks at FWHM (full width at half maximum) can be observed for the ZIF-8/PAN composites samples with decreasing reaction time, Fig. 2. This suggests that the crystallinity and crystallite size of the ZIF-8 particles, attached to the PAN nanofibers, increases with reaction time. Crystallite sizes increase from 20 to 70 nm for increasing reaction time between 30 to 90 minutes. Effects of crystallite growth and size in ZIF-8 nanopowder has previously been investigated by Tanaka *et al.*^26^

Table 1 summarizes the average crystal size of the ZIF-8 crystals along with the surface textural properties of the ZIF-8/PAN composite nanofiber and the ZIF-8 nanopowder, which are discussed in Section 3.3.

**Fig. 3:**
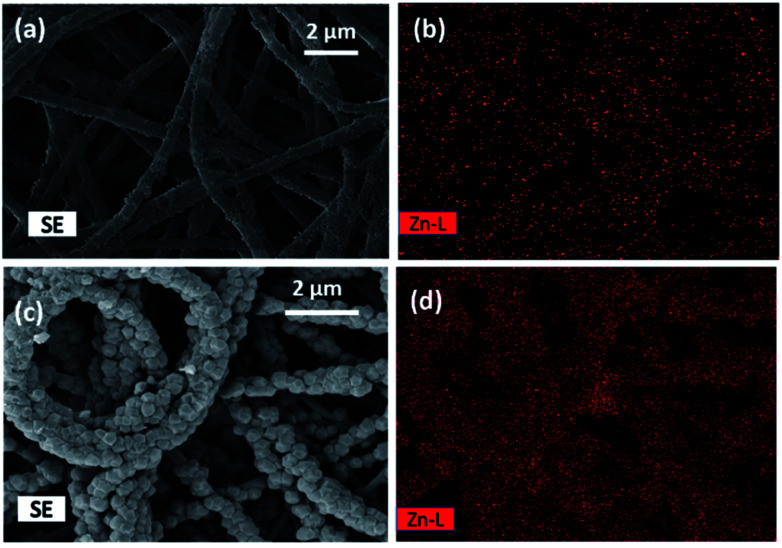
SEM micrographs and EDS mapping of Zn(OH)_2_/PAN nanofibers with (a) SEM microstructure, (b) distribution of Zn (in the top); and of ZIF-8/PAN-90 nanofibers with (c) SEM microstructure and (d) distribution of Zn (in the bottom).

Fig. 3a and c show the SEM micrographs and Fig. 3b and d the Zinc elemental mappings of the Zn(OH)_2_/PAN composite nanofibers (zinc hydroxide attached to the PAN nanofiber before the *in situ* growth of the MOF) and of the ZIF-8/PAN composite-90 nanofiber structure. The larger size and broader distribution of the Zinc in the initial Zn(OH)_2_/PAN composite (Fig. 3b) compared to the final ZIF-8/PAN-90 composite nanofiber (Fig. 3d) indicate that there occurs a redistribution of zinc atoms from the initial Zn(OH)_2_ phase during the phase conversion process into the ZIF-8 crystallites. The explanation is that during the growth of the ZIF-8 on the surface of nanofiber, zinc ions from the hydroxide phase are finally incorporated on a molecular level into the crystal structure of the MOF particles, which results in a more even and finer coverage of the PAN nanofiber surface with Zn.

### 3.2 Chemical interaction between PAN nanofiber and ZIF-8

**Fig. 4:**
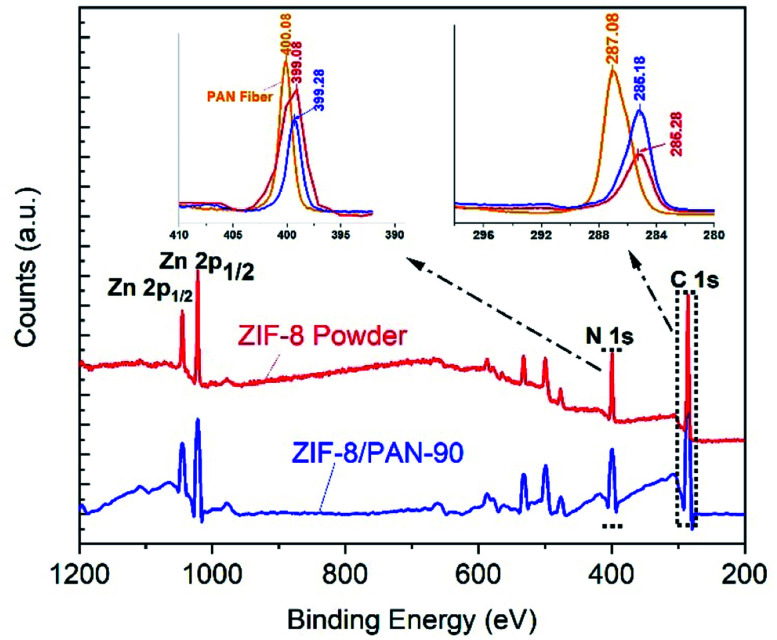
X-ray photoelectron spectroscopy (XPS) spectra of ZIF-8/PAN-90 composite nanofiber (blue) and the ZIF-8 nanopowder (red). For comparison, the XPS signals at 399 eV and 287 eV for N 1s and C 1s are zoomed in with addition of the peaks for the pure PAN fiber (in orange colour).

In order to reveal chemical interactions between the ZIF-8 phase and the PAN nanofibers, X-ray photoelectron spectroscopy (XPS) was carried out. The scan of a XPS spectrum of ZIF-8/PAN-90 and ZIF-8 powder exhibit peaks of Zn 2p_3/2_ and Zn 2p_1/2_ at 1022.08 and 1044.88 eV, respectively (Fig. 4).

To verify interaction between the PAN nanofiber and ZIF-8 framework, narrow scans of the N 1s and C 1s were analyzed^27^ for the ZIF-8/PAN-90 composite nanofiber, the ZIF-8 powder and pure PAN nanofibers, respectively (Fig. 4). The energy peak of the N 1s exhibits a 0.80 eV red shift from 400.8 to 399.3 eV after *in situ* growth of ZIF-8 on the PAN nanofiber. Similarly, the C 1s signal shows a red shift of 1.9 eV after the PAN nanofiber has been coated with ZIF-8 and resulting in ZIF-8/PAN-90 composite. These shifts are related to the change of the atomic chemical environment of carbon and nitrogen, indicating interaction between the PAN nanofiber and the ZIF-8 structure.

**Fig. 5:**
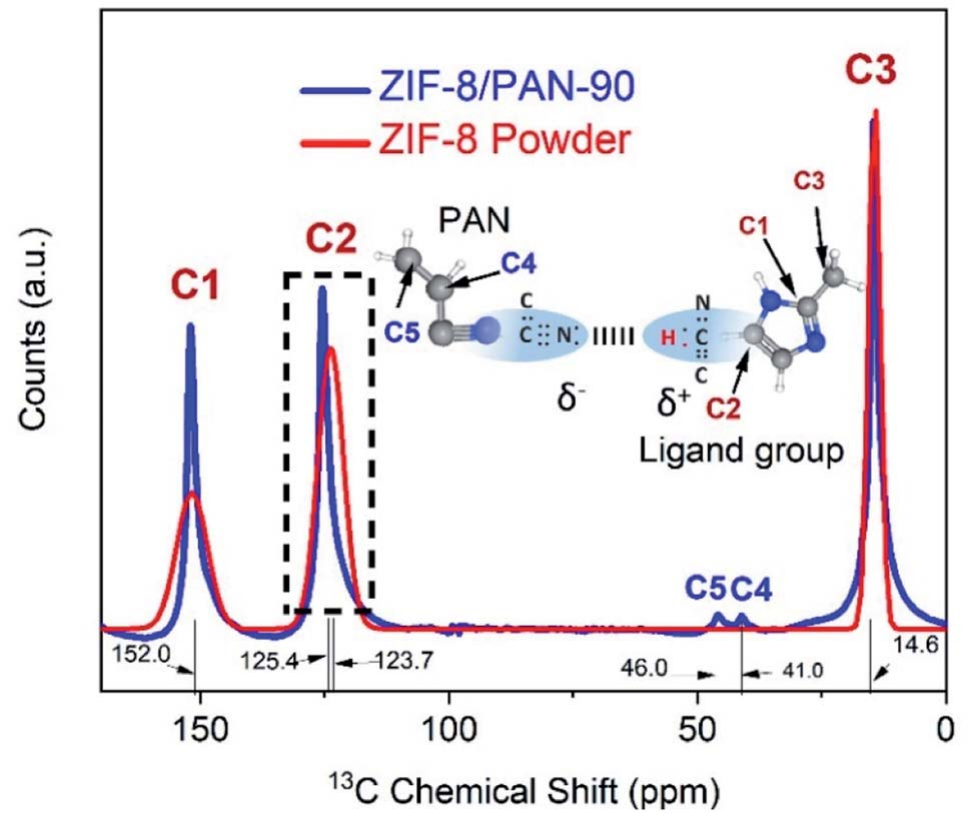
^13^C NMR spectra for ZIF-8/PAN-90 composite nanofiber (blue) and ZIF-8 nanopowder (red) and chemical formulas of PAN (nanofiber) and methyl-imidazole (organic ligand in ZIF-8) with labelling of carbons.

^13^C NMR spectroscopy was conducted to further analyse the atomic chemical environment. As shown in Fig. 5, the location of the resonance peaks at 152.0 and 14.6 ppm, respectively, can be assigned to protonated aromatic carbon atoms (C1, carbon) and the methyl group (C3, –CH_3_). These peaks have no major chemical shift.

However, there is a visible chemical shift by 1.7 ppm from 123.7 ppm to 125.4 ppm for the double-bond carbon (C2, –CH=CH–) when comparing the spectra for the ZIF-8 nanopowder with the ZIF-8 crystallites attached to the ZIF-8/PAN-90 composite nanofiber. Similar chemical shift of the C2 peak in ZIF-8 has been observed when organic molecules, such as caffeine, were encapsulated in the pores of ZIF-8 to form van der Waals bonds.^28^ However, more detailed analysis supported by DFT calculations^29^ would be required to further proof electrostatic interactions and hydrogen bonding between the ZIF-8 and C–N groups of the PAN nanofiber (as visualized by the chemical formulas in Fig. 5).

### 3.3 Surface textural properties of ZIF-8/PAN composite nanofibers

**Fig. 6:**
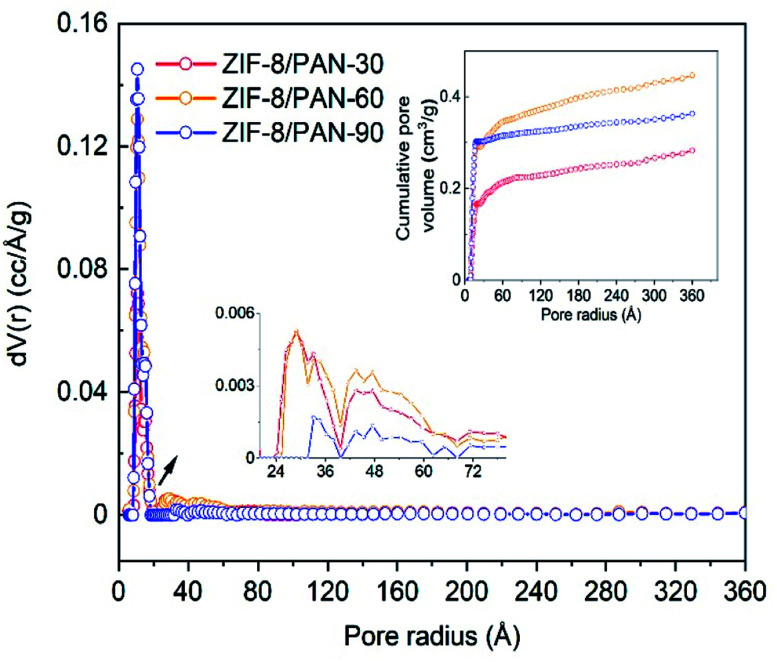
Pore size distribution and cumulative pore volume of ZIF-8/PAN composites.

Fig. 6 shows the pore size distribution of the different type of ZIF-8/PAN nanofiber structures (for the chosen synthesis time between 30 and 90 minutes).

A large peak centered around a pore radius of about 11 Å can be assigned to micropores, whereas two smaller and broader peaks between 20 to 70 A are related to micro and mesopores. Table 1 summarizes the micropore and total pore volume and BET surface area of all ZIF-8/PAN nanofibers and the ZIF-8 nanopowder. The comparably low total pore volume of 0.38 cm^3^ g^−1^ and specific surface area (495 m^2^ g^−1^) of the sample obtained after short reaction time (30 minutes), indicate that the ZIF-8 has not been fully crystallized or has major surface defects. The broad and slightly displaced XRD peaks for this sample in Fig. 2 support this thesis. After longer reaction time (90 minutes), the total pore volume increases to 0.51 cm^3^ g^−1^ and the surface area to 888 m^2^ g^−1^ for the sample ZIF-8/PAN-90. Considering a maximum ZIF-8 loading of 57.19 wt% in ZIF-8/PAN-90 achieved in this work, the surface area of the pure ZIF-8 crystallites in the composite can be calculated to 1552 m^2^ g^−1^, assuming negligible surface area of the PAN nanofibers. Using similar estimates (43 wt% PAN in the composite), the actual micropore and total pore volumes of the ZIF-8 crystallites in the ZIF-8/PAN-90 composite should be about 0.54 and 0.88 cm^3^ g^−1^, respectively. This indicates that by the *in situ* growth method proposed in this work, ZIF-8/PAN nanofiber composites with high surface area, pore volume and ZIF-8 particle loadings have been achieved compared to ZIF-8-nanofibers in other studies. Table 2, further below, gives a comparision of the CO_2_ uptake performance of the ZIF-8 nanofiber composite materials in this study compared to other studies.

**Table 2:** Comparison of ZIF-8 loading, BET surface area and CO_2_ uptake of different ZIF-8 powder and ZIF-8-nanofiber composites

Material	ZIF-8 loading (wt%)	BET surface area (m^2^ g^−1^)	CO_2_ uptake^a^ (cm^3^ g^−1^)	Reference
ZIF-8/PAN-90	57.2	888	7.0	This work
ZIF-8 powder	100	1016	14.7	This work
ZIF-8/PAN-90	57.2	888	130 (mg g^−1^, 40 bar)	This work
ZIF-8 powder	100	1813	374 (mg g^−1^, 40 bar)	Autié *et al.*^11^
ZIF-8/ZnO core–shell	—	733	7.6	Thomas *et al.*^31^
ZIF-8/PAN	—b	983	13.3 (20 °C)	Gao *et al.*^32^
ZIF-8 powder	100	880	16.5	Gao *et al.*^33^
ZIF-8 powder	100	—	15.3	Huang *et al.*^34^
ZIF-8 powder	100	1264	350 (mg g^−1^, RT, 30 bar)	Nune *et al.*^35^

a CO_2_ uptake values at low pressures are given at 25 °C, 1 bar and in cm^3^ g^−1^. If CO_2_ uptake was measured under other conditions, for example higher pressures or lower temperature, these values are given in brackets.

b The ZIF-8 loading unknown.

**Fig. 7:**
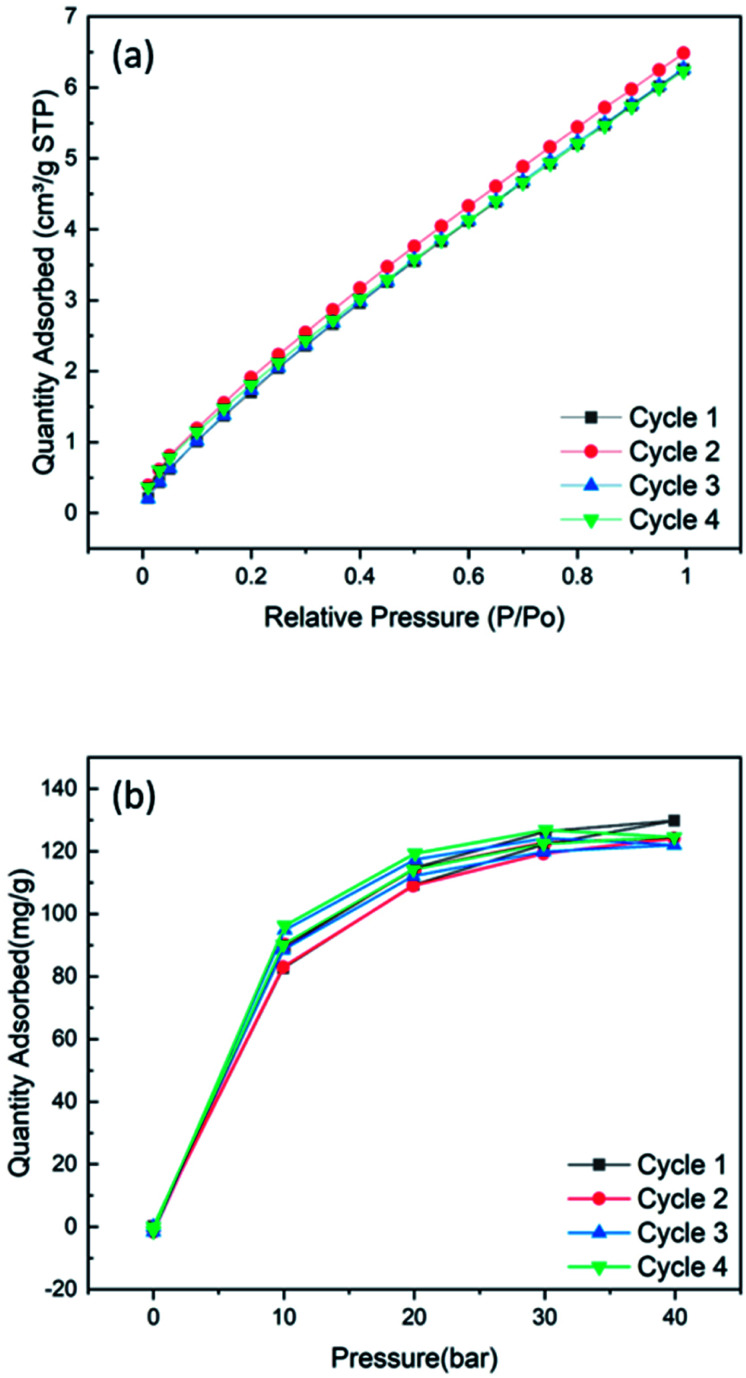
(a) Cyclic adsorption isotherms up to 1 bar at 25 °C and (b) the cyclic CO_2_ uptake of ZIF-8/PAN-90 composite at high pressure of 40 bars.

Fig. 7 shows the cyclic CO_2_ uptake and re-generation of the ZIF-8/PAN-90 at 25 °C for 1 bar and for 40 bars for four cycles in Fig. 7a and b, respectively. The CO_2_ uptake of ZIF-8/PAN-90 nanofibers at 1 bar and 40 bars is 7 cm^3^ g^−1^ and 130 mg g^−1^.

These initial results demonstrate good initial cyclic stability for CO_2_ separation with fast adsorption–desorption kinetics. Reasonable high CO_2_ uptake of ZIF-8/PAN-90 nanofiber composites have been achieved compared to similar studies on ZIF-8,^36^ suggesting that these materials are interesting for CO_2_ separation from gas streams.

## 4. Conclusion

In summary, we successfully demonstrated a phase inversion method to grow ZIF-8 crystals on the surface of a PAN polymer nanofiber matrix without sacrificing the surface properties of the material. XPS and ^13^C NMR analysis indicate electrostatic interaction and hydrogen bonds between the PAN matrix and the ZIF-8 nanocrystals confined to the PAN nanofiber surface, resulting in good attachment of the ZIF-8 nanoparticles and relatively high CO_2_ uptake. We believe that this method to grow MOF structures into polymer nanofiber structures offers further opportunities to produce nanofibrous adsorbent materials for other applications, such as air or liquid filtration or in medical applications.

## Conflicts of interest

There are no conflicts to declare.

## Supplementary Material
